# Factors Influencing Survival Status of HIV/AIDS after HAART in Huzhou City, Eastern China

**DOI:** 10.1155/2022/2787731

**Published:** 2022-10-06

**Authors:** Meihua Jin, Zhongrong Yang, Jing Li, Xiaoqi Liu, Zhenqian Wu

**Affiliations:** Huzhou Center for Disease Control and Prevention, 999 Changxing Road, Huzhou 313000, Zhejiang Province, China

## Abstract

**Background:**

Highly active antiretroviral therapy (HAART) can effectively reduce the risk of death and opportunistic infections in patients with HIV/AIDS. The aim of this study was to analyse the survival status and its influencing factors in HIV/AIDS after HAART.

**Methods:**

The data on patients' sociodemographic characteristics, treatment information, and follow-up results from the Information Management System of the Chinese Center for Disease Control and Prevention were obtained. Bivariate and stepwise multivariate Cox proportional hazards regression model analyses were performed.

**Results:**

A total of 1812 participants were included in this study, of which 1716 were still alive (survival group) and 96 had died (death group). The results indicated that respondents who were elderly (*HR* = 1.053, 95% *CI*: 1.037–1.069, *P* < 0.01), who had heterosexual transmission (*HR* = 2.422, 95% *CI*: 1.314–4.465, *P* < 0.01) and whose current WHO clinical stage was stage III or IV (*HR* = 2.399, 95% *CI*: 1.215–4.735, *P* < 0.05) were more likely to have died; respondents whose baseline CD4^+^ T-lymphocyte count was equal to or more than 200 cells/*μ*L (*HR* = 0.412, 95% *CI*: 0.275–0.616, *P* < 0.05) were unlikely to have died.

**Conclusions:**

It is recommended that HAART be provided to HIV/AIDS patients at an early clinical stage and that the health services for HIV/AIDS patients after taking medicines be strengthened, which will help promote adherence to therapeutic regimens and improve quality of life.

## 1. Introduction

Acquired immune deficiency syndrome (AIDS) is one of the major infectious diseases caused by the human immune deficiency virus (HIV) that seriously threatens human health and the safety of life and affects economic development and social stability [1–3]. Since the advent of the AIDS epidemic, no cure or effective vaccine has been found. Highly active antiretroviral therapy (HAART) is considered to be the most effective way to treat HIV/AIDS [4]. Many studies have shown that HAART plays a dual role in improving the quality of life among HIV/AIDS patients and reducing secondary infections [4, 5], and is currently recognized as the most effective way to treat patients with HIV/AIDS. HAART is a key component in the realization of the “three 90%” goals proposed by the United Nations Programme on HIV/AIDS (UNAIDS) in 2020 [5]. At the core of achieving control and reversing the epidemic trend of HIV is the idea that undetectable = untransmissible [6], and UNAIDS plans to eliminate HIV transmission by 2030 [7].

HAART can effectively reduce the mortality risk of HIV/AIDS, improve quality of life, and reduce the risk of opportunistic infections [8, 9]. The main purpose of HAART for patients is to inhibit the replication of HIV, reduce the viral load to the lower limit of detection, increase the CD4^+^ T lymphocyte count (CD4), rebuild or maintain immune function, prolong survival, and improve the quality of life [10]. The most important point of HAART is that its use among patients with HIV/AIDS greatly reduces the risk of HIV being transmitted to others.

Huzhou is an important transportation hub in China's Yangtze River Delta [11]; it is located in the North of Zhejiang Province, adjacent to Jiangsu in the East, Anhui in the West, Hangzhou in the South, and Taihu Lake (one of the four major freshwater lakes in China) in the North. With a total area of 5,818 square kilometres, the city has a population of 3.4 million. Huzhou City has implemented free HAART for HIV/AIDS since 2005. To further evaluate and grasp the effect of HIV/AIDS after HAART and understand factors influencing the survival status of HIV/AIDS patients after treatment with HAART, a retrospective cohort study was performed. This study explored the effect of relevant factors (including general demographic characteristics such as age, marriage, sex, baseline CD4 count, route of infection, current WHO clinical stage, etc.) on the mortality rate of participants after taking antiviral drugs. This study provides a scientific basis for further improving the effect of antiretroviral therapy and prolonging the survival of patients with HIV/AIDS.

## 2. Materials and Methods

### 2.1. Study Population and Data Collection

A total of 1812 cases were confirmed by the AIDS confirmation laboratory of the Huzhou Center for Disease Control and Prevention as HIV antibody-positive (both HIV antibody screening and the confirmatory test of a western blot were positive) and joined the HAART programme. The inclusion criteria for HAART were in accordance with the requirements of the “China Free HIV Antiretroviral Drug Treatment Handbook.” This study was conducted in accordance with the guidelines of the Declaration of Helsinki and approved by the Ethics Committee of the Huzhou Center for Disease Control and Prevention. Written informed consent was obtained from each participant. The history card from January 1, 2005, to December 31, 2021, was downloaded from the antiretroviral treatment module of the AIDS Comprehensive Prevention and Control Data and Information Management System of the Chinese Center for Disease Control and Prevention, and the data of the patients' basic treatment, follow-up, and CD4 cell counts were obtained.

### 2.2. Measurements

The retrospective cohort study method was adopted, and the follow-up records of HIV/AIDS after HAART in the basic information system of AIDS prevention and control of the Chinese disease prevention and control information system were collected. According to the requirements of China's free AIDS antiretroviral drug treatment manual, each HAART patient was followed up in a cohort, and the relevant epidemiological questionnaires and follow-up forms were completed. Thus, the general sociodemographic characteristics, related clinical symptoms, history of risky sexual behaviour, CD4 cell count, and other laboratory test indicators of the patients taking the drug were collected. The detection of CD4 cell count was carried out by using the FACS-Calibur instrument of BD Company, and all tests were carried out by laboratory personnel according to standard operating procedures.

### 2.3. Statistical Analysis

In this study, the censoring variable was set as the outcome variable of HIV/AIDS death, and censoring was tracked for patients who did not die as well as those who died of drug withdrawal, were lost to follow-up or referral to other places, or died of other diseases. Bivariate Cox proportional hazards regression model analyses were performed to explore the impact of relevant factors (including general demographic characteristics such as age, marital status, sex, baseline CD4 level, infection route, and current WHO clinical stage) on the mortality of study subjects after HAART. Variables with significant bivariate between-group differences were used as candidate variables in a backward likelihood ratio stepwise multivariate Cox proportional hazards regression model. All statistical analyses were performed using SPSS for Windows 19.0 and Stata 15.0, and a *P* value < 0.05 was considered to be statistically significant.

## 3. Results

### 3.1. General Demographic Characteristics and Bivariate Cox proportional Hazards Regression Model Analyses

**Figure 1:**
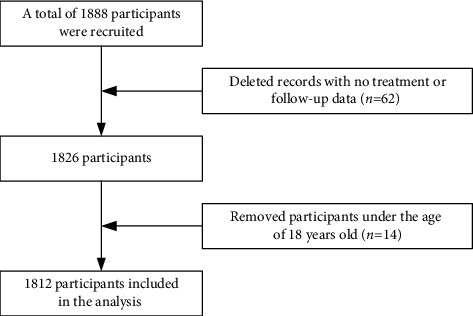
A flowchart of participants was included in the statistical analysis.

A total of 1812 participants were included in this study for analysis (Figure 1), of which 1716 were still alive (survival group) and 96 had died (death group). The mean age (mean ± SD) of the participants was 40.20 ± 14.58 years, with mean ages of 39.64 ± 14.33 years and 50.25 ± 15.55 years representing the survival group and the death group, respectively. Describing the participants in the study, it was observed that 1534 (84.7%) males, 447 (24.7%) had a primary school education and below, 858 (47.4%) were married or cohabiting marital status, 720 (39.7%) experienced sexual transmission by men who had sex with men (MSM), 1073 (59.2%) experienced sexual transmission through heterosexuality, 1537 (84.8%) were 6 months or less from confirmation of HIV positivity to joining treatment, 584 (32.2%) had a baseline CD4^+^ T lymphocyte count less than 200 cells/*μ*L, 67 (3.7%) were currently in WHO clinical stage III or IV, and 1443 (79.6%) were BMI of 18.5–24.99 (Kg/m^2^). The results of bivariate Cox proportional hazards regression model analyses showed that age, education level, route of infection, baseline CD4, and current WHO clinical stage were associated with death after HARRT (*P* < 0.05, Table 1).

### 3.2. Results of Multivariate Cox proportional Hazards Regression Model Analysis

**Figure 2:**
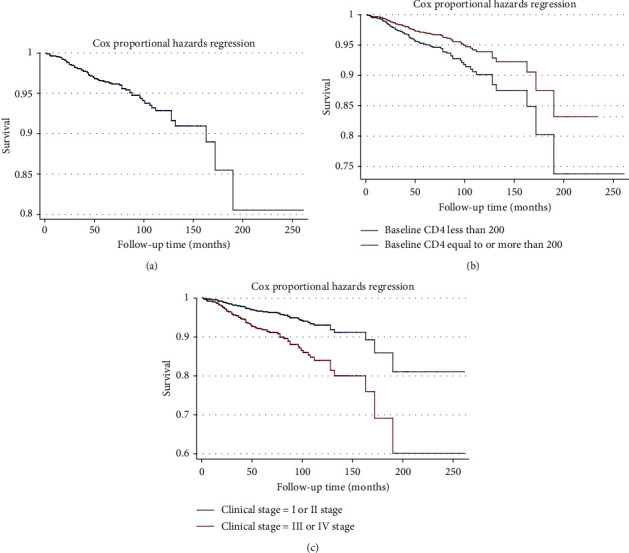
The survival curves of participating subjects: (a) overall survival curves; (b) survival curves of different baseline CD4; and (c) survival curves of different WHO clinical stages.

**Figure 3:**
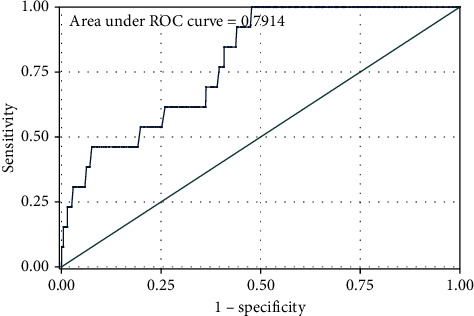
The ROC curves of predictive models in modeling groups (participants).

The variables with *P* < 0.1 in univariate analysis (including age, education level, route of infection, baseline CD4, and current WHO clinical stage) were included in multivariate Cox proportional hazards regression model analysis, and backward likelihood ratio stepwise regression analysis was performed. The results indicate that the educational level did not enter the final regression model. The results of the multivariate Cox proportional hazards regression model (Table 2) showed that respondents who were elderly (*HR* = 1.053, 95% *CI*: 1.037–1.069, *P* < 0.01), who had heterosexual transmission (*HR* = 2.422, 95% *CI*: 1.314–4.465, *P* < 0.01) and whose current WHO clinical stage was stage III or IV (*HR* = 2.399, 95% *CI*: 1.215–4.735, *P* < 0.05) were more likely have died; respondents whose baseline CD4^+^ T-lymphocyte counts were equal to or more than 200 cells/*μ*L (*HR* = 0.412, 95% *CI*: 0.275–0.616, *P* < 0.05) were less likely have died. The survival curve of the participants is shown in Figure 2. The internal validation area under the ROC curve (AUC value) obtained by this model was 0.7914 (Figure 3), indicating that the model has a good predictive effect on the risk of death after treatment for the participants.

## 4. Discussion

Huzhou City is located at the centre of the Yangtze River Delta [11] and no relevant research has been carried out in this area to date. This study has further strengthened awareness of the efficiency of HAART in treating HIV/AIDS patients and preventing transmission with its as-soon-as-possible implementation, while also emphasizing adherence to medications to improve quality of life. Studies have shown that when an HIV/AIDS partner who has had sex is timely in taking antiviral drugs afterward, their partner could be 96.0% free from HIV infection compared with others whose partners delay HAART [12]. This practice has the potential to greatly reduce the risk of HIV transmission from positive patients to the general population. In China, with the rapid popularization and promotion of the HAART strategy, especially the implementation of the “HAART as soon as it is positive found” strategy in 2016, the survival period and quality of life of HIV-infected patients have been significantly prolonged and improved [13, 14].

The results of this study suggested that the younger the patient was at the start of HAART, the lower the risk of death would be. The risk of death gradually increases with increasing age, which is consistent with a related study [15]. This may be related to the fact that autoimmune function declines faster in the elderly group; it is also more difficult for the elderly to achieve immune recovery, and older patients who start HAART are more prone to cardiovascular and neurological diseases [4, 16]. In addition, the results of this study suggested that heterosexual transmission carries a higher risk of death than MSM transmission. The main reason may be that patients with HIV/AIDS from a homosexual transmission are generally younger, and a lower age is also associated with a lower risk of developing opportunistic infections or dying [17].

The results of this study suggested that the risk of death in the group with baseline CD4 levels equal to or greater than 200 cells/*μ*L was lower than that in the group with baseline CD4 levels less than 200 cells/*μ*L. The earlier HIV/AIDS patients receive HAART, the more effective the treatment will be. Patients who have undergone HAART have shown lower infectivity, and the immune system of the patient at the time of death has basically returned to normal [18]. The earlier HAART was initiated, the better the tolerance of the drug's acute toxic side effects [19].

The new finding of this study is the ability to predict the survival curve of the participating subjects by directly using the patient's age, route of infection, base CD4^+^ T lymphocyte count, and current WHO clinical stage without using clinically relevant laboratory test indicators. The model obtained an internal validation of the area under the ROC curve (AUC value) of 0.7914 (Figure 3), suggesting that there was a good predictive effect regarding the risk of death after treatment in participating subjects.

Low CD4 levels at the start of antiviral therapy can affect immune reconstitution after treatment, and lower CD4 levels before antiviral therapy are associated with a higher risk of death. At present, the HAART standard for HIV/AIDS in China has been changed so that the patient can be treated as soon as there is confirmation of HIV positivity. This measure plays a key role in the recovery or reconstruction of the immune function of HIV/AIDS patients after early treatment and medication. At the same time, taking the drug will greatly reduce the level of HIV viral load in the patient's body, thereby not only reducing the risk of HIV infection to other people but also playing a role in preventing the development of the patient's own opportunistic infections or tumours.

The WHO clinical stage represents the severity of AIDS, with higher stages indicating a more severe disease [20]. The results of this study showed that the risk of AIDS-related death in patients with clinical stage III/IV disease was higher than that in patients with stage I/II disease. HIV-infected patients with asymptomatic signs at baseline have a lower risk of death after antiretroviral therapy, suggesting that early HAART is beneficial to maintaining and rebuilding immune function, delaying disease progression, and reducing mortality [21].

Most deaths from AIDS are due to opportunistic infections. The most common opportunistic infection in HIV/AIDS is Pneumocystis pneumonia (PCP). Patients will have symptoms such as chest tightness and coughing with fever, and most cough symptoms are not severe. However, the symptoms of chest tightness will progressively worsen, and even respiratory failure may occur, leading to death. HIV/AIDS also fosters many other opportunistic infections, such as cryptococcal meningitis and related tumours. In this study, 7 HIV/AIDS patients died from PCP, 6 HIV/AIDS patients died from non-Hodgkin's lymphoma or AIDS-related tumours, and 1 HIV/AIDS patient died from cryptococcal meningitis.

There were still some shortcomings in this study. First, this study did not advance to exploring the relationship between HAART compliance and the efficacy of HIV/AIDS control in Huzhou City, and further related research is needed. In addition, this study only included HAART data from the Huzhou area, and the sample size was relatively small. Simultaneously, due to the low number of AIDS-related deaths (*n* = 38), we included all-cause deaths in the analysis. The representativeness of the study results has certain limitations, and a larger sample of HAART data is needed to further validate the results.

Theoretically, if an HIV-infected person is relatively young and in good health when infected with HIV, with early detection and timely use of antiviral drugs, the viral load of HIV in the body is generally controlled to the extent that the virus is undetectable in the laboratory. In such cases, patients will live to the local average life expectancy. However, the antiviral treatment effect will often be affected by factors during a treatment that cause a failure to achieve this state. If the side effects are so severe that the patient cannot tolerate the drug, the treatment is eventually abandoned. Alternatively, the drug that the government or nonprofit organization provides free of charge may no longer be effective because of resistance, and the patient may not be able to afford another drug at his own expense, leading to possible interruption of treatment. These factors can affect the efficacy of treatment. However, it is certain that with timely and effective treatment, the survival time of infected people will be much higher than that of infected people without any treatment.

## 5. Conclusions

Patients in the free HAART program for HIV/AIDS in Huzhou City can improve their survival rate. Higher baseline CD4^+^ T-lymphocyte counts and a lower current WHO clinical stage reduce the risk of death. It is recommended that steps be taken to expand the monitoring of HIV high-risk groups, advocate early detection, early diagnosis, and early treatment, strengthen education regarding early compliance, and promote drug resistance monitoring among HIV/AIDS patients while administering antiretroviral therapy in a scientifically responsive manner, all facilitating adherence to treatment regimens and improving survival and quality of life for individuals living with HIV/AIDS.

## Figures and Tables

**Table 1:** General demographic characteristics and bivariate Cox proportional hazards regression model analyses.

Variables	All (*n* = 1812)	Survival group (*n* = 1716)	Death group (*n* = 96)	Univariate HR (95% CI)
	*n* (col%) or (Mean ± SD)	*n* (row%) or (Mean ± SD)	*n* (row%) or (Mean ± SD)	
Age (yrs)	40.20 ± 14.58	39.64 ± 14.33	50.25 ± 15.55	1.062 (1.047, 1.077)^*∗∗*^
Sex				
Female	278 (15.3)	262 (94.2)	16 (5.8)	1.00
Male	1534 (84.7)	1454 (94.8)	80 (5.2)	0.982 (0.573, 1.682)
Education level				
Primary school and below	447 (24.7)	405 (90.6)	42 (9.4)	1.00
Junior high school and above	1365 (75.3)	1311 (96.0)	54 (4.0)	0.381 (0.254, 0.571)^*∗∗*^
Marital status				
Married or cohabitation	858 (47.4)	809 (94.3)	49 (5.7)	1.00
Others (unmarried/divorced/widowed)	954 (52.6)	907 (95.1)	47 (4.9)	0.867 (0.581, 1.294)
Route of infection				
MSM	720 (39.7)	707 (98.2)	13 (1.8)	1.00
Heterosexual transmission	1073 (59.2)	992 (92.5)	81 (7.5)	4.336 (2.413, 7.792)^*∗∗*^
Other ways (injecting drug use, etc.)	19 (1.0)	17 (89.5)	2 (10.5)	2.226 (0.471, 10.528)
Time from confirmation of HIV positivity to joining treatment (months)				
Less than or equal to 6	1537 (84.8)	1470 (95.6)	67 (4.4)	1.00
More than 6	275 (15.2)	246 (89.5)	29 (10.5)	1.113 (0.702, 1.765)
Base CD4^+^ T lymphocyte count (cells/*μ*L)				
Less than 200	584 (32.2)	533 (91.3)	51 (8.7)	1.00
More than or equal to 200	1228 (67.8)	1183 (96.3)	45 (3.7)	0.412 (0.275, 0.616)^*∗∗*^
Current WHO clinical stage				
I or II stage	1745 (96.3)	1659 (95.1)	86 (4.9)	1.00
III or IV stage	67 (3.7)	57 (85.1)	10 (14.9)	2.098 (1.083, 4.066)^*∗*^
BMI (Kg/m^2^)				
18.5–24.99	1443 (79.6)	1371 (95.0)	72 (5.0)	1.00
<18.5	182 (10.1)	166 (91.2)	16 (8.8)	1.551 (0.901, 2.668)
25-	187 (10.3)	179 (95.7)	8 (4.3)	0.947 (0.456, 1.967)

^*∗*^ *P* < 0.05; ^*∗∗*^*P* < 0.01.

**Table 2:** Results of multivariate Cox proportional hazards regression model analysis.

Variables	*B*	SE	Wald	Sig	HR (95% CI)
Age (yrs)	0.052	0.008	43.045	0.000	1.053 (1.037, 1.069)^*∗∗*^
Route of infection					
MSM			8.046	0.018	1.00
Heterosexual transmission	0.885	0.312	8.033	0.005	2.422 (1.314, 4.465)^*∗∗*^
Other ways (injecting drug use, etc.)	0.624	0.801	0.607	0.436	1.866 (0.389, 8.965)
Base CD4^+^ T lymphocyte count (cells/*μ*L)					
Less than 200					1.00
More than or equal to 200	−0.522	0.208	6.284	0.012	0.593 (0.395, 0.892)^*∗*^
Current WHO clinical stage					
I or II stage					1.00
III or IV stage	0.875	0.347	6.362	0.012	2.399 (1.215, 4.735)^*∗*^

^*∗*^ *P* < 0.05; ^*∗∗*^*P* < 0.01.

## Data Availability

The datasets generated and analyzed during the current study are available from the corresponding authors upon reasonable request.

## Abbreviations

AIDS: Acquired immune deficiency syndrome
HIV: Human immunodeficiency virus
UNAIDS: United nations programme on HIV/AIDS
CD4: CD4^+^ T lymphocyte count
HAART: Highly active antiretroviral therapy
*HR*: Hazard ratio.
